# Frequency of Zygosity in *Jak-2* Positive Patients with Polycythemia Vera-Pakistan’s Perspective

**DOI:** 10.31557/APJCP.2021.22.2.559

**Published:** 2021-02

**Authors:** Syed Zubair Shah, Naila Raza, Muhammad Israr Nasir, Syed Mustanir Hussain Zaidi

**Affiliations:** 1 *Department of Hematology, Liaquat National Hospital and Medical College, Karachi, Pakistan. *; 2 *Department of Molecular Pathology, Liaquat National Hospital and Medical College, Karachi, Pakistan. *; 3 *Department of Statistics, Liaquat National Hospital and Medical College, Karachi, Pakistan. *

**Keywords:** Arms, PCR, Zygosity, mutational load, polycythemiaVera, JAK2V617F mutation, Pakistan

## Abstract

**Background:**

Estimation of *JAK2V617F* mutational load in Polycythemia Vera (PV) helps to determine the severity of the disease phenotype, the risk of thrombotic events, progression to post-PV myelofibrosis and survival. Amplification Refractory Mutation Screening (ARMS) PCR or Allele Specific (AS) PCR is a simple easy method with a reasonable sensitivity for screening of zygosity.The purpose of this study was to see the frequency of disease burden and phenotypic characteristics in Pakistani patients diagnosed with *JAK2V617F* mutation positive PV.

**Materials and Methods:**

A cross-sectional study using non probability consecutive sampling was conducted at Hematology Department, Liaquat National Hospital Karachi from October 2018 to July 2019.Adult newly diagnosed *JAK2V617F *positive PV patients of either gender were included. Patients’ demographics, clinical characteristics and baseline CBC were noted. JAK2V617F zygosity was qualitatively analyzed by ARMS-PCR technique. Age and gender were stratified to see to see the result of qualitative and quantitative effect modifiers on these patients using Chi Square and fisher exact test as appropriate while mean comparison was done by independent t-test and one way ANOVAtest. P value of ≤0.05 was considered as significant.

**Results:**

Fifty one patients were included in the study with an average age of 59.60±14.29years.90.2% of patients had hypertension.All patients tested positive for heterozygous state. Significant association of gender was found with smoking (p=0.001) while age was significantly linked with hypertension (p-0.033).

**Conclusion:**

*JAK2V617F *positive PV patients are mainly heterozygous males showing significant association with smoking and hypertension. ARMS-PCR is a robust technique to determine zygosity which can be used for screening purposes.

## Introduction

Polycythemia vera (PV) is a clonal proliferative disorder, categorized under BCR-ABL negative Myeloproliferative Neoplasm (MPN). About 95% of the patients demonstrate recurrent mutation in the Janus tyrosine kinase 2 (*JAK2*) gene, consisting of a valine-to-phenylalanine change at position 617 (JAK2 617V>F) in the JH2 pseudo-kinase domain (Campbell and Green, 2006). This mutation in cytokine-dependent cell lines provides cytokine independence and increased sensitivity through the activation of STAT5, Akt and ERK-dependent pathways (Dupont et al., 2007; Cross, 2011;Passamonti et al., 2011). However, recent research has shown that the clinical heterogeneity of MPNs is probably closely linked to their genetic complexity as many other genetic or epigenetic abnormalities in addition to JAK2V617F were detected in this group(Vainchenker et al., 2011; Saeidi, 2016). *JAK2V617F* mutation detection comprises part of the diagnostic criteria of MPN based on WHO classification (Swerdlow, 2017).Studies have indicated that estimation of* JAK2V617F* mutational loadhelps to determine the severity of the disease phenotype, the risk of thrombotic events, progression to post-PV myelofibrosis and survival (Vannucchi et al., 2008; Koren-Michowitz et al., 2012). Thus, quantification of the *JAK2V617F* mutation at diagnosis not only gives vital prognostic information but is also very useful in monitoring response to therapy with JAK2V617F inhibitors, alpha-interferon, or allogeneic stem-cell transplantation (Bjørn et al., 2014).

The *JAK2V617F* mutation can be present in a heterozygous state or can progress to homozygosity most frequently by a mitotic recombination event causing uniparentaldisomy (Godfrey et al., 2012). Many studies have used the term Homozygous when mutational load is more than 50% and Heterozygous when it is less than 50% (Tefferi et al., 2006; Vannucchi et al., 2007; Malysz and Crisan, 2009). In west, two highly sensitive molecular techniques; quantitative polymerase chain reaction (qPCR) and droplet digital PCR (ddPCR) are currently available for a quantitative evaluation of *JAK2V617F *mutation allele burden in patients diagnosed with MPNs (Guglielmelli et al., 2017). However, these tests are costly and mainly confined to reference laboratories. ARMS-PCR or Allele Specific (AS) PCR is a simple and easy method,it permits a single base change to be detected under ideal PCR conditions.This testis capable of detecting low levels of mutations with a reasonable sensitivity of 1% to 5%; hence mutational load of greater than 50% suggests homozygosity and less than 50% heterozygous state (Link-Lenczowska et al., 2018).

This study was conducted to see the frequency of disease burden and phenotypic characteristics in Pakistani patients diagnosed with *JAK2V617F* mutation positive PV. No data is available locally and to our knowledge this study is the first from Pakistan. 

The objective of our study was to:

• Qualitatively assess the frequency of *JAK2V617F *mutational load in PV patients. 

• Look for association of zygosity with disease phenotype at presentation. 

## Materials and Methods

This is a cross sectional study conducted at Department of Haematologyand Molecular Pathology, Liaquat National Hospital and Medical College, Karachi from October 2018 to July 2019 after approval from ethical review committee of Liaquat National Hospital Karachi and taking informed consent from patients. Sample size was calculated by WHO software for sample size calculator as minimum 50 patients. Newly diagnosed adult *JAK2V617F* mutation positive PV patients as per WHO criteria of either gender were included in the study(Swerdlow, 2017). Patients younger than 18 years or with MPN other than PV or having secondary causes of Polycythemia were excluded.* JAK2V617F* mutation zygositywas qualitatively analyzed by ARMS-PCR technique (Chen et al., 2007). Data was collected by researcher himself ensuring confidentiality. The effect modifiers and biases were strictly controlled by inclusion and exclusion criteria.

*Extraction of total genomic DNA from whole peripheral blood*

Blood samples were collected from each subject in 5cc vacutainer tubes containing 1.8 mg/ml K2 EDTA. DNA isolation from peripheral blood was performed using a DNAzol™ BD Reagent (Invitrogen, Catalog number: 10974020) according to manufacturer’s procedure. The concentration and quality of DNA was checked using Qubit 2.0 Fluorometer and QubitdsDNA BR Assay Kit (Invitrogen, Catalog number: Q32853).

*ARMS-PCR*

**Figure 1:**
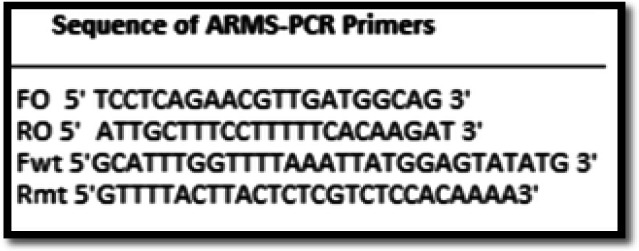
Primer Sequence Used in ARMS-PCR (Chen et al., 2007)

ARMS-PCR assay uses two primer pairs to specifically amplify the normal (229 bp) and mutant (279 bp) sequence (two tube assay). A 100 ng of DNA template and HotStarTaq Master Mix Kit (Cat # 203443) were used for the amplification. Sequence for primers and amplification were adapted (Chen et al., 2007) (Figure 1). Mismatches were included in primers to maximize discrimination of the wild-type and mutant alleles. Thermal cycling conditions were initial denaturation at 94 °C for 1 minute (min), 40 cycles of denaturation at 95°C for 30seconds(s), annealing at 58 °C for 40 s and extension at 72 °C for 45 s. Final extension was done at 72°C for 10 min. Products were resolved on 2 % agarose gel for 45 min at 120 V with 100 bp ladder and visualized under ultraviolet illuminator after staining with Ethidium Bromide.

*Zygosity Labelling*

Heterozygous *JAK2V617F +ve* mutation when amplification of 279bp (mutant) and 229bp (normal) was seen.

Homozygous* JAK2V617F +ve* mutation when amplification of 279bp was detected.

Sensitivity was established using JAK2V617F reference standard 50% (Horizon cat # HD649) with further dilution in JAK2 wild type reference standard (Horizon cat # HD652). 

*Statistical Analysis*

Mean and standard deviation were computed for quantitative variable and frequency and percentage were calculated for qualitative variables. Stratification was done with regards to qualitative variables to see the effect of these modifiers on gender and age group by using chi square test and Fisher’s exact test. Mean comparison was done by independent t-test and ANOVA.P value≤0.05 were considered as significant. All of the statistical analyses were carried out by using SPSS software version 25.0 for Windows (SPSS Inc., IL, USA).

## Results

A total of 51 JAK2V617Fpositive patients were enrolled in the study. The results showed that there were 33 male and 18 female patients. The mean age of patients was 59.60±14.29 years. Majority (90.2%) of patients had hypertension, 35.3% were smokers. 49% had splenomegaly and only one third (29.4%) presented with thrombosis. Mean hemoglobin, Hematocrit, Total leucocyte count, Platelets, Serum Lactate Dehydrogenase and phenotypic characteristics are shown in Table 1.

**Figure 2:**
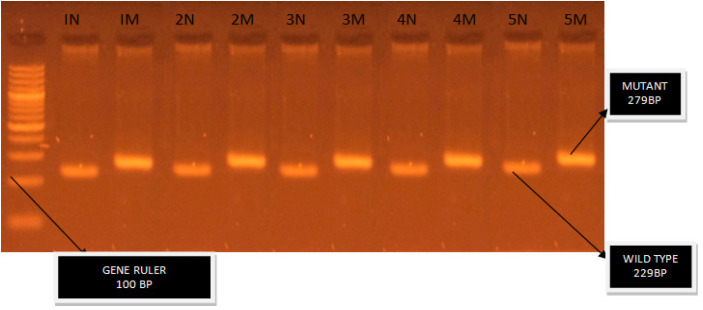
Gel Image Showing N in Each Lane/Case Represents Normal Allele. M in each lane /case represent mutant allele (Total cases 5).

The ARMS-PCR test showed that all 51 patients confirmed their positivity for heterozygous state by displaying a mutant band of 279 bp and a wild type band of 229 bp (Figure 2). 

Comparison of phenotypic characteristics with genotype (homo- and heterozygous groups) was not possible so we looked for association between cardiovascular risk factors i.e. smoking, HTN, DM and splenomegaly with gender and age stratified groups. 

Significant association of gender was found with smoking (p=0.001) while age was significantly associated with hypertension (p=0.033). We also found significant mean PLT difference with gender (p=0.021). Significant mean hemoglobin difference was also found with age group (p=0.041). Detailed results are presented in Table 2 and Table 3.

**Table 1:** Descriptive Statistics of Study Population

Variables	Value
Age (years) (Mean±SD)	59.60±14.29
Hemoglobin (g/dl) (Mean±SD)	18.21±1.74
Hematocrit (%) (Mean±SD)	55.43±5.65
Total Leucocyte Count (×10^9^/L)(Mean±SD)	17.65±10.90
Platelets (×10^9^/L) (Mean±SD)	502.17±284.01
Serum Lactate Dehydrogenase Level (U/L) (Mean±SD)	417.29±119.48
Gender	
Male (%)	33 (64.7)
Female (%)	18 (35.3)
Hypertension	
Yes (%)	46 (90.2)
No (%)	5 (9.8)
Diabetes Mellitus	
Yes (%)	20 (39.2)
No (%)	31 (60.8)
Smoking	
Yes (%)	18 (35.3)
No (%)	33 (64.7)
Splenomegaly	
Yes (%)	25 (49)
No (%)	26 (51)
Thrombosis	
Yes (%)	15 (29.4)
No (%)	36 (70.6)

**Table 2:** Descriptive Statistics of Study Population According to Gender

Variables	Value		P-Value
	Male	Female	
Hemoglobin(g/dl) (Mean±SD)	18.37±1.94	17.92±1.29	0.384º
Hematocrit (%)(Mean±SD)	54.84±6.65	56.22±2.99	0.318º
Total Leucocyte Count(×10^9^/L) (Mean±SD)	16.36±11.51	20.01±9.53	0.257º
Platelets(×10^9^/L) (Mean±SD)	435.15±252.12	625.05±304.75	0.021º
Serum Lactate Dehydrogenase Level (U/L) (Mean±SD)	425.03±118.72	403.11±123.00	0.537º
Hypertension			
Yes (%)	30 (90.9)	16 (88.9)	1
No (%)	3 (9.1)	2 (11.1)	
Diabetes Mellitus			
Yes (%)	12 (36.4)	8 (44.4)	0.572
No (%)	21 (63.6)	10 (55.6)	
Smoking			
Yes (%)	17 (51.5)	1 (5.6)	0.001
No (%)	16 (48.5)	17 (94.4)	
Splenomegaly			
Yes (%)	16 (48.5)	9 (50)	0.918
No (%)	17 (51.5)	9 (50)	
Thrombosis			
Yes (%)	10 (30.3)	5 (27.8)	0.85
No (%)	23 (69.7)	13 (72.2)	

ºIndependent t test was applied; Chi-Square test was applied; P≤0.05, considered as significant.

**Table 3:** Descriptive Statistics of Study Population According to Age Groups

Variables	Value				P-Value
	≤40 years	41-50 years	51-60 years	>60 years	
Hemoglobin(g/dl) (Mean±SD)	17.58±1.82	16.82±2.05	18.82±1.19	18.55±1.61	0.041º
Hematocrit (%)(Mean±SD)	56.50±4.68	52.00±5.63	57.76±4.44	55.44±5.96	0.197º
Total Leucocyte Count (×10^9^/L) (Mean±SD)	10.11±2.64	13.45±12.02	16.66±6.81	20.78±11.80	0.088º
Platelets(×10^9^/L) (Mean±SD)	530.00±305.67	387.12±165.63	472.00±238.73	538.78±319.82	0.598º
Serum Lactate Dehydrogenase Level (U/L) (Mean±SD)	474.83±79.11	457.12±121.80	441.66±150.09	385.75±111.01	0.204º
Hypertension					
Yes (%)	4 (66.7)	6 (75)	9 (100)	27(96.4)	0.033
No (%)	2 (33.3)	2 (25)	0 (0)	1 (3.6)	
Diabetes Mellitus					
Yes (%)	4 (66.7)	1 (12.5)	3 (33.3)	12 (42.9)	0.228
No (%)	2 (33.3)	7 (87.5)	6 (66.7)	16 (57.1)	
Smoking					
Yes (%)	3 (50)	1 (12.5)	3 (33.3)	11 (39.3)	0.47
No (%)	3 (50)	7 (87.5)	6 (66.7)	17 (60.7)	
Splenomegaly					
Yes (%)	5 (83.3)	5 (62.5)	3 (33.3)	12 (42.9)	0.205
No (%)	1 (16.7)	3 (37.5)	6 (66.7)	16 (57.1)	
Thrombosis					
Yes (%)	1 (16.7)	1 (12.5)	4 (44.4)	9 (32.1)	0.528
No (%)	5 (83.3)	7 (87.5)	5 (55.6)	19 (67.9)	10

˚ANOVA was applied; Fisher exact test was applied; P≤0.05, considered as significant.

## Discussion

Since the discovery of* JAK2V617F* mutation specific to MPNs in 2005 it has been integrated as an essential diagnostic test. The utility of estimating the allelic burden is now been explored to assess the prognosis and response to therapy.This study aimed at screening the *JAK2V617F* mutation positive PV patients for disease burden; whether homo- or heterozygous using ARMS-PCR technique.

The most interesting finding of our study was that the entire cohort tested positive for heterozygous status based on qualitative analysis. Data from West have reported the presence of homozygous status in approx 25-30% cases (Baxter et al., 2005; Levine et al., 2005; Campbell and Green, 2006), Comparison with regional studies shows a wide variationin mutational burden among PV patients.The heterozygous state has been reported as 47% from Sudan, 46% from India and 91% from Egypt among PV patients (Ayad and Nafea, 2011; Abkar et al., 2017; Sazawal et al., 2019). Remarkably identical results as ours i.e. 100% heterozygous state were reported by Ferdowsi et al., (2016) from Iran among a cohort of 86 MPN patients using similar techniques. Presence of heterozygosity in our cohort can be coincidental as the sample size was small, due to demographic changes between continents or low sensitivity of the method used.

Perricone et al., (2017a) compared the qualitative ARMS-PCR methodto the quantitative approach noted thatboth techniques detected a mutationalburden of 1% but high variability was seen in the former technique on repeated sampling (Perricone et al., 2017a). Many recent studies have shown that a small clonal hematopoiesis may be present in otherwise healthy subjects at low levels (0.03–1%) (Martinaud et al., 2010; Nielsen et al., 2013). A recently published study concluded that the detection of a *JAK2V617F* mutation at low levels is debatable to be labeled as MPN (Perricone et al., 2017b). Also the current British Society Guidelines suggests that assays sufficiently sensitive to detect a mutational load as low as 1–3% should be used (McMullin et al., 2019). Based on the above-mentioned studiesit can be inferred that a reasonable presumption of zygositycan be made using the robust ARMS-PCR technique.

Another noteworthy result of this study was that 65% of the tested cohort were males all having heterozygous disease. It is contradictory to other studies which have reported women as having lower mutational burden than men (Stein et al., 2010; Godfrey et al., 2012). We found significant association of HTN and smoking among cardiovascular risk factors. On age stratification HTN was found to be notably linked with our test population (p value=0.033) and 100% patients in age bracket 51 to 60 years had HTN. This findingconcurs withmultiple studies reported earlier (Tefferi et al., 2013; Horvat et al., 2019).Similarlysmoking was prevalent among males (p value=0.001); a predictable finding as two thirds of the tested patients was males and smoking in females is uncommon in South Asian culture.

Our results did not show considerable association between thrombosis and heterozygous state. Thisresultalso endorsesmultiple studies which have linked thrombosis at diagnosis or follow up with a very high mutational burden of 75-100% (Borowczyk et al., 2015; Bertozzi et al., 2017; Zhang et al., 2020).

This study lacked disease stratification based on zygosity as all patients turned out to be heterozygous; nevertheless, it can be considered as the first endeavor towards assessing the genotypic pattern of JAK2V617F positive PV with disease characteristics among Pakistani patients.

In conclusion, our data show that in Pakistan JAK2V617F positive PV patients are mainly heterozygousmales showing significant association with smoking and hypertension.ARMS-PCR is a robust technique which can be used for screening purposes. More studies are needed for accurate estimation of allele burden by quantitative analysis and to develop a better understanding of the disease phenotype and outcome. 

## Limitations

Major drawback of this study was that only qualitative assessment was done and the sample size was small. For quantification of allelic burden more sensitive and accurate techniques such as quantitative real-time PCR is recommended for future studies.
